# A Bayesian Inferential Approach to Quantify the Transmission Intensity of Disease Outbreak

**DOI:** 10.1155/2015/256319

**Published:** 2015-02-15

**Authors:** Adiveppa S. Kadi, Shivakumari R. Avaradi

**Affiliations:** Department of Studies in Statistics, Karnatak University, Dharwad 580003, India

## Abstract

*Background*. Emergence of infectious diseases like influenza pandemic (H1N1) 2009 has become great concern, which posed new challenges to the health authorities worldwide. To control these diseases various studies have been developed in the field of mathematical modelling, which is useful tool for understanding the epidemiological dynamics and their dependence on social mixing patterns. *Method*. We have used Bayesian approach to quantify the disease outbreak through key epidemiological parameter basic reproduction number (*R*_0_), using effective contacts, defined as sum of the product of incidence cases and probability of generation time distribution. We have estimated *R*_0_ from daily case incidence data for pandemic influenza A/H1N1 2009 in India, for the initial phase. *Result*. The estimated *R*_0_ with 95% credible interval is consistent with several other studies on the same strain. Through sensitivity analysis our study indicates that infectiousness affects the estimate of *R*_0_. *Conclusion*. Basic reproduction number *R*_0_ provides the useful information to the public health system to do some effort in controlling the disease by using mitigation strategies like vaccination, quarantine, and so forth.

## 1. Introduction

Influenza is an emerging infectious disease and influenza-like illness (ILI) is a clinical illness caused by the influenza virus, which gave rise to human pandemics such as 1918 Spanish flu (H1N1), 1957 Asian flu (H3N2), 1968 Hong Kong flu (H2N2), and most recently H1N1 pandemic 2009. Influenza A (H1N1) was originally referred to as “swine flu” because laboratory testing showed that many of the genes in this new virus were very similar to those found in pigs in North America. Further on, it has been found that this new virus has gene segments from the swine, avian, and human flu virus genes. The scientists call this a “quadruple reassortant” virus and hence this new (novel) virus is christened “influenza-A (H1N1) virus” [1].

A reassorted influenza was first detected in Mexico on March 18, 2009, and rapidly spread to the United States, Canada, and subsequently all regions worldwide including India. The first case of H1N1 was reported in India on May 17, 2009, at Hyderabad Airport in a young boy who travelled from the USA and later it spread throughout the country [2]. According to World Health Organization (WHO) update of November 20, 2009, virus spread across more than 206 countries resulting in 6770 deaths [3]. In June 2009 World Health Organization (WHO) raised the level of pandemic alert phase 5 to phase 6 [4]. According to the Directorate General of Health Services, Government of India, New Delhi, update on November 19, 2009, there have been 16044 laboratories confirming cases recorded in India resulting in 537 deaths [5].

To formulate the valid and reliable estimate of transmissibility and spread of an outbreak we have utilised statistical modelling, which facilitates our understanding of mechanism of disease spread. To access the intensity of an outbreak, transmission potential can be quantified by reproduction number *R*_0_, that is, average number of secondary cases generated by a single primary case in a completely susceptible population [6, 7]. The importance of basic reproduction number *R*_0_ becomes more apparent when an emerging infectious disease strikes a population which is a key concept in the epidemic theory. If *R*_0_ is less than or equal to one, then transmission in the population goes stochastically extinct with probability of one after a small number of infections. If *R*_0_ is greater than 1, then there is a positive probability of a large epidemic. Statistical estimation of *R*_0_ is used to understand the transmission dynamics and evolution of the infectious disease which facilitate designing the effective public health intervention strategies and mitigation policies [8].

The aim of present study is to quantify the intensity of pandemic influenza A/H1N1 2009 in India. To achieve this goal we have calculated basic reproduction number from time series data set of H1N1 through Bayesian approach to contact patterns. We have estimated basic reproduction number *R*_0_ with 95% credible interval.

## 2. Material and Methods

### 2.1. Data Sources

**Figure 1:**
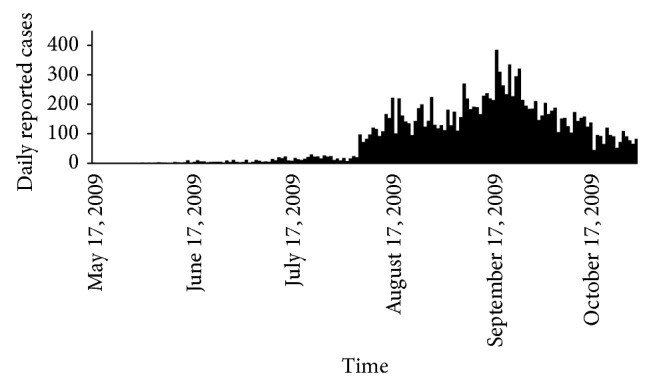
Daily reported cases of influenza A/H1N1 2009 of India.

Time series data for the 2009 influenza A/H1N1 pandemic in India was obtained from the Ministry of Health and Family Welfare (MoHFW), Government of India [9]. 31,924 infected cases with 1525 deaths were reported during May 17, 2009, to May 17, 2010, with two complete waves of epidemic. We have represented the data of the first wave from May to October 2009 (see Figure 1). For the analysis we have used daily reported cases (incidence) for initial phase of an epidemic from June 11 to August 13, 2009 [10].

### 2.2. Statistical Method

Statistical inference of *R*_0_ is still in progress, and it is recognized that the estimate is very sensitive to dispersal of the disease progression [11]. Estimation of *R*_0_ can be illustrated by employing time-since-infection model and suggests origin of transmission of infectious diseases which is the counterpart of compartmental models like SI, SIS, and so forth. Both models are originated in the basic paper of Kermack and McKendrick [12], and both the SIR model and the simplest time-since-infection model are known as “the Kermack-McKendrick age structure model.” It is used to identify key epidemiological parameter by using a simple renewal process which adheres to the basic reproduction number *R*_0_.

Let *j*(*t*) represent the number of new infections or incidence at calendar time *t*; that is, each infected individual on an average generates secondary cases at a rate *A*(*τ*) at time-since-infection *τ* which is a specific case of renewal equation of birth process [13–15]. Consider

(1) $$\begin{array}{c} j\left(t\right)=\overset{\infty }{\underset{0}{\int }}A\left(\tau \right)j\left(t-\tau \right)d\tau . \end{array}$$

Since *R*_0_ represents average number of secondary cases that a primary case generates during entire his/her infectious period, the estimate is given by [16, 17]

(2) $$\begin{array}{c} R_{0}=\overset{\infty }{\underset{0}{\int }}A\left(\tau \right)d\tau . \end{array}$$

Under Kermack and McKendrick assumption “single infection causes an independent process of infection with host” which allows an age representation for the state of infection, that is, infectivity of an individual. The time elapsed since infection is called the infection age or time since infection, whereas *A*(*τ*) is expected infectivity of an individual with time-since-infection *τ* whereas *A*(*τ*) becomes the rate of secondary transmission per single primary case at time-since-infection *τ* [18]:

(3) $$\begin{array}{c} A\left(\tau \right)=\beta \left(\tau \right)\Gamma \left(\tau \right), \end{array}$$

where *β*(*τ*) is the transmission rate which depends on frequency of contact and infectiousness at infection age *τ* and Γ(*τ*) is the probability of being infectious at infection age *τ*. Substituting *A*(*τ*) into (1) we get

(4) $$\begin{array}{c} j\left(t\right)=\overset{\infty }{\underset{0}{\int }}\beta \left(\tau \right)\Gamma \left(\tau \right)j\left(t-\tau \right)d\tau . \end{array}$$

Further, we consider a probability density of the generation time where generation time is defined as time from infection of an individual to the infection of a secondary case by that individual, denoted by *g*(*τ*) through normalized density of secondary transmission [16, 17, 19]. We have

(5) $$\begin{array}{c} g\left(\tau \right)=\frac{\beta \left(\tau \right)\Gamma \left(\tau \right)}{\int_{0}^{\infty }\beta \left(\tau \right)\Gamma \left(\tau \right)d\tau }=\frac{\beta \left(\tau \right)\Gamma \left(\tau \right)}{R_{0}}. \end{array}$$

Using (5) in (4) we get

(6) $$\begin{array}{c} j\left(t\right)=\overset{\infty }{\underset{0}{\int }}R_{0}g\left(\tau \right)j\left(t-\tau \right)d\tau . \end{array}$$

Then, the basic reproduction number *R*_0_ is as in

(7) $$\begin{array}{c} R_{0}=\frac{j\left(t\right)}{\int_{0}^{\infty }g\left(\tau \right)j\left(t-\tau \right)d\tau }. \end{array}$$

In reality, the case incidences are rather in discrete form as daily/weekly reports. The discretized analogy of (7) can be derived as

(8) $$\begin{array}{c} R_{0}=\frac{j_{i}}{\sum_{s=0}^{\infty }w_{s}j_{i-s}}. \end{array}$$

By taking the inverse of both sides of (8) we get

(9) $$\begin{array}{c} \frac{1}{R_{0}}=\frac{\sum_{s=0}^{\infty }w_{s}j_{i-s}}{j_{i}}, \end{array}$$

where *s* is the discretized form of time since infection (usually in days), *i* is discretized calendar time (usually in days/weeks), and *w*_*s*_ is the generation time distribution for an infectious disease which is the probability distribution function for the time from infection of an individual to the infection of a secondary case by that individual [19, 20].

**Figure 2:**
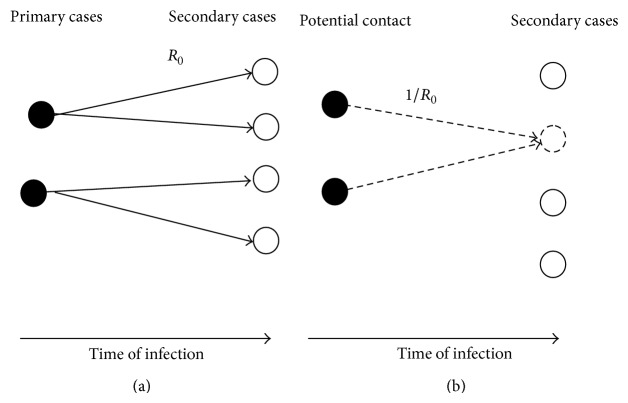
Transmission tree for contact patterns.

From Figure 2 the concept of *R*_0_ through contact patterns has been illustrated here before taking up the Bayesian method of estimation. From Figure 2(a), the transmission tree with *R*_0_ = 2 represents who infected whom, where each primary case on an average generates “2” secondary cases. Secondary transmissions from primary to secondary cases are given by the basic reproduction number *R*_0_ = 2. From Figure 2(b) restructuring the transmission tree, given that all the potential contacts made by primary cases with the probability of each possible contact resulted in a secondary transmission, is 1/*R*_0_. This type of transmission tree represents who infected whom which is unobservable in nature unless rigorous contact tracing is implemented [21, 22].

The numerator of the right hand side of (9) represents the total number of effective contacts made by a possible primary case in day/weeks which have an equal probability of resulting in the secondary transmission; that is, the probability that a secondary case is linked to an effective contact made by a single primary case at time *i* is given by 1/*R*_0_. Effective contacts lead to potential secondary cases with equal chance of getting the infection from the primary cases at time *i*. It has been seen that every contact does not lead to successful transmission of infection; that is, the effective contacts are uncertain which is defined as the contact that is sufficient to lead to the transmission of infection between infectious and susceptible population. The total number of effective contacts made by a potential primary case at time *i* is the sum of the product of incidence cases and generation time distribution during the generation interval of length *m*. This indicates a simple binomial law of uncertainty in effective contacts [22].

Total number of effective contacts become *c*_*i*_ = ∑_*s*=0_^*m*^ *w*_*s*_ *j*_*i*−*s*_, during the *i*th day, that is, *c*_*i*_ ~ Bin(*j*_*i*_, 1/*R*_0_), ∀*i* = 1, 2,…, *n*, where 1/*R*_0_ is the probability of effective contacts. Let *j*_*i*_ denote incidence or new cases at calendar time *i* and *w*_*s*_ denotes generation time distribution at time-since-infection *s*. Then the probability mass function of effective contacts becomes

(10) Pci1R0=jici1R0Ci1−1R0ji−ci,∀i=1,2,…,n; 0<1R0<1.

The likelihood function of 1/*R*_0_ is as follows:

(11) L1R0⋱Ci=∏i=1njiCi1R0Ci1−1R0ji−CiL1R0=∏i=1nji∑s=0mwsji−s ·1R0∑s=0mwsji−s1−1R0ji−∑s=0mwsji−s.

Here, Bayesian inferential approach is used to estimate parameter *R*_0_ which provides us with different but related estimate by combining prior belief and the evidence observed. As more evidence is gathered the prior distribution is modified into the posterior distribution that represents the uncertainty over the parameter values. Posterior distribution is derived from the Bayes formula [23]

(12) $$\begin{array}{c} \pi \left(\theta /x\right)=\frac{f\left(x/\theta \right)\pi \left(\theta \right)}{\int_{\Theta }^{\cdot }f\left(x/\theta \right)\pi \left(\theta \right)d\theta }, \end{array}$$

where ∫_Θ_^·^ *f*(*x*/*θ*)*π*(*θ*)*dθ* is a normalization constant, *x* indicated data, *θ* is the unknown quantity, *π*(*θ*) is the prior distribution, *f*(*x*/*θ*) is the likelihood function, and posterior distribution *π*(*θ*/*x*) completely describes the uncertainty. There are two key advantages of Bayesian theory: (i) once the uncertainty in the posterior distribution is expressed via probability distribution then the statistical inference can be automated and (ii) available prior information is reasonably incorporated into the statistical model. Now, the posterior estimate of parameter *R*_0_ is derived using its prior information, where likelihood function follows binomial distribution with conjugate prior as beta distribution of first kind [24]. That is, 1/*R*_0_ ~ Beta(*a*, *b*), with realistic choice of parameters (*a*, *b*):

(13) π1R0=1β(a,b)1R0a−11−1R0b−10<1R0<1, a>0, b>0.

Now posterior distribution of 1/*R*_0_ is proportional to the likelihood times prior. Therefore,

(14) Posterior=likeliHood∗priornormalization  constatnt=∏i=1njici1/R0∑i=1nci1−1/R0∑i=1nji−∑i=1nci1/βa,b1/R0a−11−1/R0b−1∫1/R0·∏i=1njici1/R0∑i=1nci1−1/R0∑i=1nji−∑i=1nci1/βa,b1/R0a−11−1/R0b−1d(1/R0)=1/R0∑i=1nci+a−11−1/R0∑i=1nji−∑i=1nci+b−1∫011/R0∑i=1nci+a−11−1/R0∑i=1nji−∑i=1nci+b−1d1/R0=Beta∑i=1nci+a,∑i=1nji−∑i=1nci+b.

**Figure 3:**
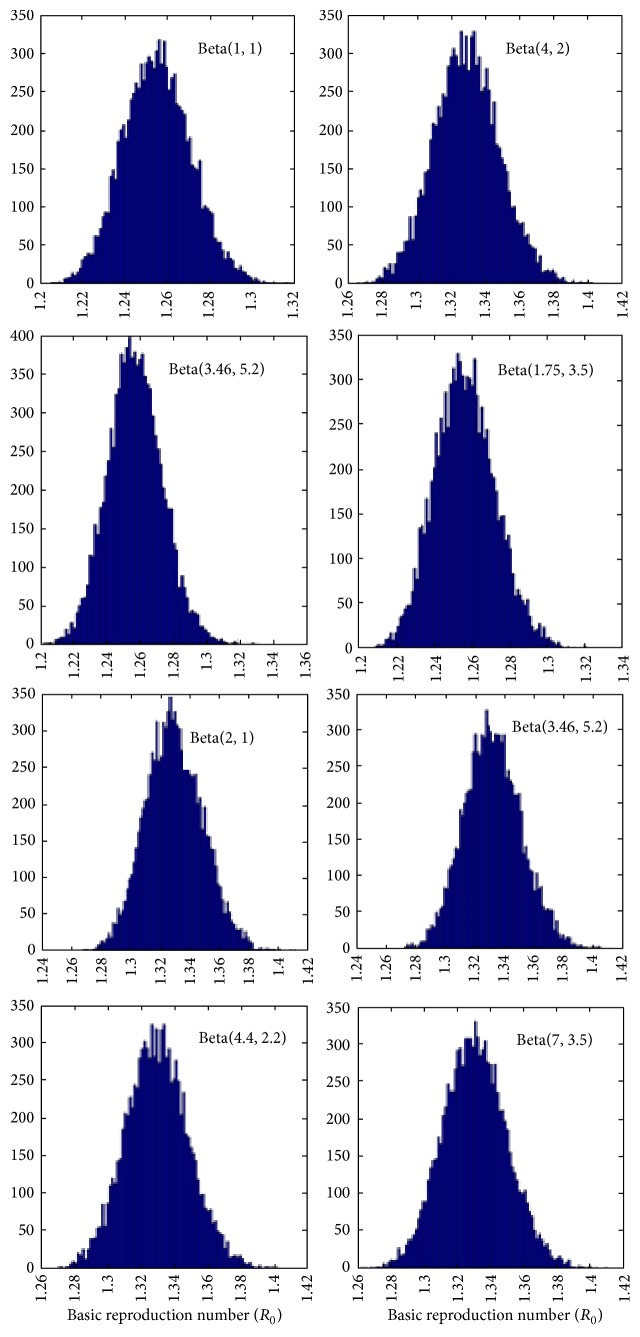
Histogram of posterior distribution of *R*_0_ by using different values of prior choices for beta distribution.

We are interested in estimating *R*_0_ with its 95% credible interval (CrI) which has been derived by considering the sampling transformation of 1/*R*_0_ through simulation from the above posterior distribution with different choices of priors for beta distribution. Theoretically, it is hard to find the posterior distribution of *R*_0_ where 1/*R*_0_ is a beta variable. Through simulation we have generated 10000 samples from beta posterior distribution and also estimated 95% CrI. We have considered beta distribution with several combinations of mean (ranges from 0.4 to 0.8) and accordingly we chose different values of (*a*, *b*). The second and fourth column of Table 1 represent our estimates of *R*_0_ which are posterior means along with 95% CrI.  Figure 3 displays the posterior distribution of *R*_0_ with different prior choices.

Generation time is another most important characteristic in infectious disease epidemiology, since *R*_0_ indicates only the average number of secondary infections one primary infection produces in one disease generation. When we consider disease transmission in real time scale such as days or weeks, it matters a lot how long one disease generation lasts. Generation time is the average time taken for secondary infections produced by a primary infection [25]. Generation interval or generation time distribution is assumed to be known as Weibull distribution which is a biologically plausible choice [20, 26–28] with a mean of 1.78 and 2.48 days and a standard deviation (SD) of 0.66 and 1.06 days for *s* = 7 and 10 days [29, 30] (Tables S1 and S2 in Supplementary Material available online at http://dx.doi.org/10.1155/2015/256319). All the sensitivity analysis was done by using MATLAB (Supplementary Material, Algorithm).

### 2.3. Sensitivity Analysis

Sensitivity analysis is a statistical technique which provides insight into how uncertainty in input variables affects the model outputs and which input variable tends to derive variation in the outputs [31]. We performed sensitivity analysis to quantify the effect of changes on *R*_0_. It has been used to determine how sensitive an estimate of the parameter is. It is usually performed as series of tests in which one can use different set of hyperparameter values to see the change in the estimate. Our analysis is based on the pandemic influenza A/H1N1 in India 2009 through the Bayesian estimates of basic reproduction number; we used the daily reported cases to calculate effective contacts. We have calculated posterior distribution of *R*_0_ using prior as beta distribution with different values of parameter choices. From Figure 1 we have seen that as prior choice changes the shape of the posterior distribution also changes.

## 3. Results and Discussion

The estimates of *R*_0_ for the 2009 H1N1 influenza pandemic were mainly reported based on the data obtained in the first few months of pandemic or based on whole first wave data. Most of these *R*_0_ estimates ranges from 1.1 to 2 [32–37]. Our estimated value of the basic reproduction number indicates the milder intensity of disease transmission in India. Interestingly, this estimated *R*_0_ with 95% credible interval is consistent with several other studies on the same strain [35], along with many European countries [28]. Notably, it has a smaller credible length which is more reliable estimate; see Table 1. Statistical inference of *R*_0_ is based on incidence (reported cases) and known generation time distribution. Some differences among these estimates are due to the choice of generation time distribution because *R*_0_ estimation relies much on the assumptions of the generation time distribution [38]. In general, shorter mean generation time may lead to smaller *R*_0_ estimates. Since, the estimate of *R*_0_ crucially depends on generation time distribution. From Table 1, we conclude that generation time or infectiousness of an individual affects the basic reproduction number. This method does not require exponential growth assumption. Still our estimate is greater than one so one has to make effort in controlling the disease through control strategies, which are typically targeted to bring this number below one and maintain it, as this will lead to eventual extinction of the epidemic.

### 3.1. Limitations

This method is applied only for initial stage of the epidemic (exponential phase) when there is no intervention like quarantine, isolation vaccination, and so forth. If basic reproduction number is *R*_0_ < 1, then the probability 1/*R*_0_ terminates because it exceeds the law of probability.

## Supplementary Material

We have calculated mean and standard deviation (SD) of the generation time distribution with different values of shape and scale parameters (See table S1), similarly we have evaluated mean of the prior distribution with different choice of hyper parameters whose mean ranges from 0.4 to 0.8 for 7 days and 10 days.(see table S2). Estimation of basic reproduction number R_0_ as well as sensitivity analysis was done through simulation using MATLAB. (Tables S1 and S2 in Supplementary Material available online at ).

## Figures and Tables

**Table 1:** Sensitivity analysis of basic reproduction number R_0_ is depending on generation time distribution as Weibull distribution for time since infection *s* for 7 days as well as 10 days.

Prior distribution for *s* = 7 days	Basic reproduction number R_0_ (with 95% CrI)	Prior distribution for *s* = 10 days	Basic reproduction number R_0_ (with 95% CrI)
Beta(1,1)	**1.2548** (1.2223, 1.2923)	Bata(2,1)	**1.3392** (1.3128, 1.3938)

Beta(4,2)	**1.2543** (1.2250, 1.2850)	Beta(3.46,5.2)	**1.3323** (1.2962, 1.3762)

Beta(3.46,5.2)	**1.2578** (1.2309, 1.2909)	Beta(4.4,2.2)	**1.3296** (1.2895, 1.3695)

Beta(1.75,3.5)	**1.2569** (1.2279, 1.2879)	Beta(7,3.5)	**1.3303** (1.2969, 1.3669)
