# Supplementary material for: A Bayesian Inferential Approach to Quantify the Transmission Intensity of Disease Outbreak
Source: Comput Math Methods Med. 2015 Feb 15;2015:256319. doi: 10.1155/2015/256319 (PMC4345055; doi:10.1155/2015/256319)
Supplement: Supplementary file 1 — We have calculated mean and standard deviation (SD) of the generation time distribution with different values of shape and scale parameters (See table S1), similarly we have evaluated mean of the prior distribution with different choice of hyper parameters whose mean ranges from 0.4 to 0.8 for 7 days and 10 days.(see table S2). Estimation of basic reproduction number R0 as well as sensitivity analysis was done through simulation using MATLAB. (Tables S1 and S2 in Supplementary Material available online at ). [file 256319.f1.pdf]

Supplementary Information:

Weibull distribution is used as generation time distribution for 7 days and 10 days

Probability density function is as follows:

$$f_{\lambda,\alpha}(x) = \alpha\lambda^{\alpha}x^{\alpha-1}e^{-(\lambda x)^{\alpha}} \qquad x > 0, \alpha - shape\ and\ \lambda - scale$$

With mean and variance

$$mean = \frac{1}{\lambda}\Gamma(1 + \frac{1}{\alpha})$$

$$variance = \frac{1}{\lambda^2}\left[\Gamma\left(1 + \frac{2}{\alpha}\right) - \Gamma^2\left(1 + \frac{1}{\alpha}\right)\right]$$

Table S1: Mean and standard deviation of generation time distribution

Generation Time Distribution				
	**λ**	**α**	**Mean**	**Standard Deviation (SD)**
**7 days**	2.8	2	1.78	0.66
**10 days**	2.5	2.8	2.48	1.06

Prior distribution is beta distribution which is given below:

$$\pi(x) = \frac{1}{\beta(a,b)} \quad (x)^{a-1}(1-x)^{b-1} \qquad 0 < x < 1, \qquad a > 0, b > 0$$

Table S2: Mean of the Prior distribution

Prior Distribution (Beta)			
	a	***b***	**Mean**
**7 days**	1	1	0.5
	4	2	0.66
	3.46	5.2	0.39
	1.75	3.5	0.33
	2	1	0.66

**10 days**	3.46	5.2	0.39
	4.4	2.2	0.66
	7	3.5	0.66

Simulation algorithm:

To perform Monte Carlo simulations of contact patterns to calculate posterior distribution with estimate of basic Reproduction Number R_0. In this article we use the concept of effective or potential contacts described in Nishiura H 2010[] to calculate effective contacts we need daily reported cases and Generation time distribution. We have used Weibull distribution for generation time distribution for infectious period of 7 days and 10 days. Briefly,

1. Create Probability distribution function of weibull distribution (w_s) for $s = 7$ days with parameters$(\lambda = 2.8, \alpha = 2)$.

2. Calculating cross product of daily cases(j_{i-s}) and PDF created in step 1 we get effective contacts as $\sum_{s=0}^{\infty} w_s j_{i-s}$

3. By using prior distribution as beta distribution with different parameter choices, we have calculated posterior distribution which is beta distribution where as likelihood is binomial distribution.

4. To get the estimate of R_0 :

i) Generate 10000 random numbers from posterior distribution (Beta distribution)

ii) Taking inverse of random numbers, we get posterior distribution of R_0

iii) All these numbers are arranged in the frequency distribution table using **histc( )** function

iv) Estimated R_0 using the observation in step (iii)

v) Calculated credible interval (upper and lower) using **Find()** function in MATLAB.

Same process was done for Weibull distribution (w_s) for $s = 10$ days with parameters($\lambda = 2.5, \alpha = 2.8$)
